# Understanding the associations between maternal high-risk fertility behaviour and child nutrition levels in India: evidence from the National Family Health Survey 2015–2016

**DOI:** 10.1038/s41598-022-20058-1

**Published:** 2022-10-22

**Authors:** Milan Das, Arup Jana, T. Muhammad

**Affiliations:** International Institute for Population Sciences (IIPS), Mumbai, India

**Keywords:** Nutrition, Public health

## Abstract

Anthropometric markers are the most important aspect of a child’s health assessment. Using large-scale nationally representative data from the National Family Health Survey (NFHS-4), 2015–2016, this study aimed to investigate the relationship between children born to women with high-risk fertility behaviours and children’s health outcomes. The sample consisted of 2,55,726 children of currently married women aged 15–49 years in India. The key explanatory variable, high-risk fertility behaviour was defined by women’s age at birth (below 18 or above 34 years), birth interval (less than 24 months), and higher birth orders (four and above). The key outcome variables for assessing child health outcomes were stunting, wasting, and underweight in children aged 0–59 months. We used descriptive statistics, Pearson’s chi-square test and logistic regression models to analyse the objectives. Approximately 33% of children were born with any single high-risk condition in the last 5 years in India. The bivariate analysis showed that all three components of child health, stunting, wasting, and underweight, were higher among children born to women with high-risk fertility behaviour. The findings from the multivariable analysis suggest that children born with a high risk fertility behaviour were suffering from stunting (AOR = 1.30; 95% CI 1.27–1.33) and underweight (AOR = 1.23; 95% CI 1.20–1.27). In addition, children born to women of multiple high-risk categories had higher odds of stunting (AOR = 1.53; 95% CI 1.46–1.59) and underweight (AOR = 1.38; 95% CI 1.32–1.44) as compared to children born to women with no risk. Our findings highlight an urgent need for effective legislation to prevent child marriage that would be helpful in increasing the maternal age at birth. The government should also focus on the interventions in health education and improvement of reproductive healthcare to promote optimal birth spacing.

## Introduction

Childhood malnutrition is one of the most important universal public health concerns in resource-limited countries like India, a chronic impediment to a healthy life, and a constant threat to the development of human capital^1,2^. Despite the global burden of malnutrition declining, around 149 million children were still stunted, and 45 million were wasted in 2020^3^. Past evidence suggests that undernutrition puts children at a higher risk of dying from childhood illness and severe morbidity^4–6^. It contributes to 45% of fatalities in under-5 years of children^7^. A study predicted that more than 60 countries will fail to achieve the third goal of the Sustainable Development Goals (SDGs), which is to eradicate preventable newborn deaths by 2030^8^. In this context, India has the worst performance in the prevalence of underweight children, approximately double that of Sub-Saharan Africa^9^. In 2015–2016, 38%, 21%, and 36% of Indian children were stunted, wasted, and underweighted, respectively^10^.

There is a lack of evidence and a clear appraisal as to why child malnutrition is higher in India despite having a higher Human Development Index (HDI)^11^. In the World Hunger Index (WHI), neighboring countries such as Sri Lanka, Nepal, Bangladesh, Myanmar, and Pakistan are ranked 64th, 73rd, 75th, 78th, and 88th, respectively, much better than India (94th rank among 107 countries)^12^. Moreover, India loses up to 4% of its Gross Domestic Product (GDP) and 8% of its products owing to child malnutrition^13^. While several studies have highlighted poverty as the primary driver of undernutrition^14,15^, increased GDP has not translated into significant changes in nutritional status among children in India.

Previous research has shown that low socioeconomic status, unhealthy habits, rapid urbanization, and lack of education are mainly responsible for malnutrition in India^16,17^. But, past studies have not been focused on high-risk fertility behaviour such as pregnancy in adolescence, having more than three children, < 24 months birth interval, and giving birth at an older age (> 35 years) as significant risk factors of malnutrition^18,19^. Despite being the world's second-most populous country, the total fertility rates (TFRs) declined from 3.4 children per woman to 2.1 children per woman between 1992–1993 and 2015–2016 in India. Nevertheless, it is a source of concern in India that more than one-fourth of women aged 20–24 years were married before they turned 18 years. Moreover, 27% of children were born within 24 months of their parents’ last birth^10^.

Previous research has shown that a short birth interval negatively impacts both the child’s and mother’s nutritional status^20,21^. It has been observed that exhausting the mother’s nutrient supply can increase the risk of intrauterine growth obstruction and have an adverse impact on the baby’s nutrition store at birth^21^. Moreover, almost a quarter of reproductive-age women are malnourished in India^22^. Similarly, recent studies reported that the son preference still exists in India^23^. As a result, increasing birth order can occur due to unintended pregnancy, leading to neglect of child care by their parents^24^. As the number of children increases, the probability of antenatal care, postnatal care, and newborn checkups decreases^25,26^. In addition, previous evidence established that being married during adolescence or at a young age at birth has adversely been associated with the child's nutritional status^21,27^. Even if the infant does not have a high birth order or short birth interval, the study indicated that giving birth at young or older age increases the likelihood of the child having a low nutritional status^28^. Thus, an in-depth study is necessary to examine the single and combined impact of high fertility risk on a child's nutritional status. The Indian government has established a variety of nutrition-related programmes and strategies such as the Integrated Child Development Services (ICDS), Janani Suraksha Yojana (JSY), Reproductive Child Health (RCH) programme, Mid-day Meal (MDM), National Food Security Mission (NFSM), and so on to combat the malnutrition^29^. Regardless of the national program, India is unable to improve child nutrition levels.

It is worth noting that high-risk fertility behaviour is a significant predictor of maternal chronic undernutrition^30^. Moreover, women who suffer from chronic malnutrition are more likely to give birth to children with malnutrition, which causes the cycle of malnutrition for generations^31^. Previous studies have discovered that low socioeconomic status, limited access to health care and education, a lack of antenatal visits, and unwanted pregnancies are the leading causes of high-risk fertility behaviour and create a precondition for child malnutrition^32–34^. Thus, an in-depth study on the link between high-risk fertility behaviours and malnutrition among children under 5 years could help India achieve the second SDG. The main goal of this study was to look into the role of high-risk maternal behaviour in chronic under-nutrition such as stunting, wasting, and underweight among children under the age of five.

## Methods

### Data source

The present study used secondary data from India's National Family and Health Survey (NFHS-4) 2015–2016. The NFHS is a cross-sectional, nationally representative sample survey designed to provide information on population maternal and child health, fertility-related behaviour, and anthropometric measurements. The NFHS is a large-scale survey coordinated by the International Institute for Population Sciences (IIPS) under the Ministry of Health and Family Welfare (MoHFW), Government of India. The NFHS is a demographic health survey conducted in India as part of the global demographic and health survey (DHS) program. The NFHS survey has been done in four rounds by IIPS: NFHS-1 in 1992–1993, NFHS-2 in 1998–1999, NFHS-3 in 2005–2006, and NFHS-4 in 2015–2016. The NFHS-4 used a stratified two-stage sample design to collect data. The NFHS obtained information from a nationally representative sample of households as well as men, women, and children. Detailed data collection procedures are available on the DHS website. The NFHS-4 included a representative sample of 601,509 households, out of which 699,686 women aged 15–49 years were interviewed and 259,627 births in the last 5 years. In terms of data extraction, we considered children under the age of five and compiled data on high-risk fertility behaviour as well as the anthropometric measures of the children. After the data cleaning, the final sample size for the study was 145,270 mothers-children’s pairs who were included in the final analysis.

### Outcome variables

For this study to examine the association between high-risk fertility behaviours and child health, we defined stunting, wasting, and underweight as the children whose height-for-age Z-score, wasting is defined as weight for height Z score and weight for age Z score is below minus two standard deviations (-2SD)^35^.

The main independent variable was the maternal high-risk fertility behaviour, defined by the following criteria^33^:

- *High-risk fertility behaviour* women who gave birth at birth less than 18 or above 34 years old, birth interval less than 24 months, or birth order 4 and higher.
- *Single high-risk fertility behaviour* when a woman reported to have one high-risk fertility behaviour, she either gave birth either at a younger age of less than 18 years, or above 34 years, or at a birth interval less than 24 months, or high-birth order (four and above).
- *Multiple high-risk fertility behaviour* when a woman had a combination of at least two of the above-mentioned behaviours.

### Control variables

The other control variables were selected based on previous studies^13,36–39^. The age of the child was classified as 0–11, 12–23, 24–35,36–47 and 48–59 years. The gender of the child was classified as male and female, and the child disposal stool was classified as safe and unsafe. The maternal age was classified as 15–24, 25–34 and 35–49. Maternal educational attainment was divided into four categories: no education, primary, secondary and higher. Maternal Body Mass Index (BMI) was classified as underweight, normal, and overweight, and contraception use was recoded as no and yes. The number of household members in a household was classified into three categories: 1–4, 5–6, and 7 + . The wealth status of the households was obtained from the wealth quantile, calculated using the household amenities^10^. The households’ religious beliefs were recoded as the Hindu, Muslim, and Others. The caste of the household’s head was classified as Schedule Cast (SC), Schedule Tribe (ST) and Others. Sources of drinking water were categorized into ‘improved’ and ‘unimproved’ following the WHO/UNICEF definition^40^. The place of residence was recoded as urban and rural. Six geographical regions, covering 28 states and 5 UTs, were included in the analysis. These regions were classified into six: North (Jammu & Kashmir, Himachal Pradesh, Punjab, Rajasthan, Chandigarh, Uttara hand, Haryana, and Delhi), Central (Uttar Pradesh, Chhattisgarh, and Madhya Pradesh), East (West Bengal, Jharkhand, Odisha, and Bihar), North-East (Sikkim, Arunachal Pradesh, Nagaland, Manipur, Mizoram, Tripura, Meghalaya, and Assam), West (Gujarat, Maharashtra, Goa, Dadra & Nagar Haveli and Daman & Du), and South (Andhra Pradesh, Telangana, Karnataka, Kerala, Tamil Nadu, and Puducherry).

### Statistical analysis

Descriptive analyses were performed, and the results were presented in the form of unweighted frequencies and weighted percentages. Bivariate analyses (cross-tabulations) were carried out to examine the distribution of the covariates according to nutritional outcomes in children like stunting, wasting, and underweight. Also, the distribution of the nutritional outcomes was examined based on the distribution of the child born in the various high-risk categories. Pearson's chi-square statistic was used to examine the outcomes of descriptive statistics. In order to find out the association between the nutritional outcomes of the children and high-risk births, unadjusted and multivariable logistic regression analyses were conducted. The variables which were significant at *p* < 0.05 (which was considered enough to control residual confounding in the multivariable model) in the crude analysis were included in the multivariable logistic regression analyses. Both unadjusted odds ratios (UORs) and adjusted odds ratios (AORs) were reported with 95% confidence intervals (CIs). All the analyses were conducted using STATA 14.0. The estimates were based on appropriate sampling weights.

### Ethics declaration

After filing a request for the data access form, measure DHS granted ethical clearance. This study makes use of publicly available secondary data that is aggregated and does not include any personal identifiable information that can be linked to study participants. The data was considered secret and were anonymized.

## Results

### Background characteristics of the respondents

Table 1 provides the sample sizes by background characteristics. About 54% of the sample's children were male, while female children make up the remaining 46%. In this study, 57% of the mothers were between 25 and 34 yaers of age, and about 27% of the mothers were illiterate. Approximately 64% of the sample hailed from low-income households, and 80% of the sample believed in the Hindu religion. About 64% of the families disposed of potentially unsafe child’s stools and 73% of the households were obtained their water from unprotected sources. The overwhelming of the study's children roughly 70% came from rural areas. The central region was where almost 27% of the sample inhabited. Any type of high-risk fertility behaviour was evident in 35% of births. Almost, 9.4% of newborns had a birth interval of less than 24 months.

**Table 1:** Sample size with background characteristics of children aged 0–59 years, India NFHS-4.

Variables	Frequency	Percentage
**Current age of the child (months)**		
0–11	28,584	19.4
12–23	28,620	19.8
24–35	28,116	19.3
36–47	30,704	21.1
48–59	29,296	20.3
**Gender of the child**		
Male	77,938	53.9
Female	67,382	46.1
**Maternal age**		
15–24	45,257	33.2
25–34	83,362	57.1
35–49	16,701	9.7
**Maternal education**		
No education	40,672	27.1
Primary	20,183	13.2
Secondary	68,381	47.2
Higher	16,084	12.5
**Maternal body mass index**		
Under weight	35,986	25.7
Normal	87,743	58.1
Overweight	21,591	16.2
**Contraceptive use**		
No	79,224	52.3
Yes	66,096	47.7
**Number of households members**		
01-Apr	36,373	25.9
05-Jun	52,692	36.1
7 +	56,255	38.0
**Wealth**		
Poor	96,443	63.6
Non-poor	48,877	36.4
**Religion**		
Hindu	1,09,076	81.2
Muslim	18,096	13.6
Others	18,148	5.2
**Caste**		
Scheduled Caste/Tribes	57,666	32.8
Others	87,654	67.3
**Source of drinking water**		
Protected	37,475	26.7
Unprotected	1,07,845	73.3
**Child stool disposal**		
Safe	52,745	36.3
Unsafe	92,575	63.8
**Place of residence**		
Urban	37,262	29.6
Rural	1,08,058	70.4
**Region**		
North	23,482	12.5
Central	44,824	27.3
East	30,501	25.3
North east	20,881	3.3
West	10,473	13.0
South	15,159	18.6
**Any high-risk category**		
No	95,592	65.0
Yes	49,728	35.0
**Single high-risk category**		
Births to mothers < 18 years	3348	2.7
Births to mothers > 34 years	2815	1.5
Births born < 24 months	13,182	9.4
Births with a birth order > 4	14,065	8.7
**Multiple high-risks categories**		
Age at birth < 18 years and birth interval < 24 months	195	0.2
Age at birth > 34 years and birth interval < 24 months	135	0.1
Age at birth > 34 years and birth order > 4	4458	2.4
Age at birth > 34 years, birth internal < 24 months, and birth order > 4	531	0.3
Birth interval < 24 months and birth order > 4	3704	2.3
Total	1,45,320	100

### Prevalence of stunting, wasting, and underweight by socioeconomic characteristics

The prevalence of stunting, wasting, and underweight was shown in Table 2 in addition to the 95% Confidence Interval (CI) for each background characteristic. Nearly 36%, 21%, and 34% of children of those subject to mothers any high-risk fertility behaviour were stunted, wasted, and underweighted, respectively. Additionally, for children exposed to multiple high-risk fertility behaviours, 54% and 48% of children suffered from stunting and underweight. Compared to female children, male children were more suffered from malnutrition. The prevalence of stunting (45%) and wasting (27%), as well as underweight (48%), was greater in the offspring of underweight mothers. The results showed that children of scheduled caste and scheduled tribe mothers were higher rates of stunting (42%) and wasting (24%) and underweight (41%). Children having unimproved drinking water made up around two-fifths of those who were stunted and underweight. The rate of stunting was 42% in the central region of India, whereas it was only 29% in the south.

**Table 2:** Prevalence of children nutrition outcomes by sociodemographic characteristics and high-risk births among children aged 0–59 months, India NFHS-4.

Variables	Stunting (%)		Wasting (%)		Underweight (%)	
	Prevalence	95% CI	Prevalence	95% CI	Prevalence	95% CI
**Any high-risk category**						
No	37.4	(37.1–37.7)	22.0	(21.7–22.2)	35.5	(35.2–35.8)
Yes	35.9	(35.5–36.3)	21.3	(20.9–21.6)	34.2	(33.8–34.6)
*p*-value	< 0.001		0.3		< 0.001	
**High-risk category**						
No risk	33.4	(33.1–33.7)	21.7	(21.5–22.0)	32.2	(31.9–32.5)
Single risk	44.2	(43.7–44.7)	21.7	(21.3–22.2)	41.5	(41.0–42.0)
Multiple risks	53.7	(52.7–54.7)	22.0	(21.1–22.9)	47.7	(46.6–48.7)
*p*-value	< 0.001		0.875		< 0.001	
**Current age of the child (months)**						
0–11	21.3	(20.8–21.7)	29.9	(29.3–30.4)	27.5	(27.0–28.0)
12–23	41.1	(40.5–41.6)	21.9	(21.5–22.4)	33.8	(33.3–34.4)
24–35	40.9	(40.3–41.5)	19.8	(19.3–20.3)	37.0	(36.4–37.6)
36–47	42.1	(41.6–42.7)	18.8	(18.4–19.2)	38.1	(37.6–38.6)
48–59	38.5	(38.0–39.1)	18.6	(18.2–19.1)	38.6	(38.0–39.1)
*p*-value	< 0.001		< 0.001		< 0.001	
**Gender of the child**						
Male	37.7	(37.3–38.0)	22.4	(22.1–22.7)	35.7	(35.4–36.0)
Female	36.0	(35.6–36.3)	20.9	(20.6–21.2)	34.3	(34.0–34.7)
*p*-value	< 0.001		< 0.001		< 0.001	
**Maternal age**						
15–24	35.3	(34.9–35.8)	23.5	(23.1–23.9)	34.5	(34.0–34.9)
25–34	36.6	(36.3–37.0)	20.8	(20.6–21.1)	34.6	(34.3–34.9)
35–49	43.8	(43.1–44.6)	21.0	(20.4–21.6)	39.9	(39.2–40.7)
*p*-value	< 0.001		< 0.001		< 0.001	
**Maternal education**						
No education	50.2	(49.7–50.7)	23.6	(23.2–24.1)	46.8	(46.3–47.3)
Primary	42.7	(42.0–43.4)	22.3	(21.7–22.9)	40.7	(40.1–41.4)
Secondary	31.9	(31.5–32.2)	21.3	(21.0–21.6)	31.0	(30.6–31.3)
Higher	20.8	(20.2–21.4)	18.7	(18.1–19.3)	19.2	(18.5–19.8)
*p*-value	< 0.001		< 0.001		< 0.001	
**Maternal body mass index**						
Under weight	45.1	(44.6–45.6)	27.4	(27.0–27.9)	47.5	(47.0–48.0)
Normal	36.2	(35.9–36.5)	21.2	(20.9–21.5)	33.3	(33.0–33.7)
Overweight	26.3	(25.7–26.9)	14.7	(14.2–15.2)	21.6	(21.1–22.1)
*p*-value	< 0.001		< 0.001		< 0.001	
**Contraceptive use**						
No	37.3	(36.9–37.6)	23.1	(22.8–23.3)	35.8	(35.5–36.2)
Yes	36.5	(36.1–36.9)	20.3	(20.0–20.6)	34.3	(33.9–34.6)
*p*-value	0.154		< 0.001		< 0.05	
**Household members**						
1–4	34.7	(34.2–35.2)	22.2	(21.8–22.6)	33.4	(32.9–33.9)
5–6	37.3	(36.8–37.7)	21.9	(21.6–22.3)	35.7	(35.3–36.1)
7 +	38.1	(37.6–38.5)	21.2	(20.9–21.6)	35.6	(35.2–36.0)
*p*-value	< 0.001		< 0.05		< 0.001	
**Wealth**						
Poor	43.4	(43.1–43.7)	23.2	(23.0–23.5)	41.4	(41.1–41.7)
Rich	25.6	(25.2–26.0)	19.1	(18.8–19.4)	24.1	(23.7–24.4)
*p*-value	< 0.001		< 0.001		< 0.001	
**Religion**						
Hindu	36.9	(36.6–37.2)	22.1	(21.9–22.4)	35.4	(35.2–35.7)
Muslim	39.0	(38.3–39.7)	20.1	(19.5–20.7)	35.2	(34.5–35.9)
Others	30.9	(30.2–31.5)	19.8	(19.2–20.4)	29.0	(28.3–29.6)
*p*-value	< 0.001		< 0.001		< 0.001	
**Caste**						
Scheduled Caste/Tribes	41.7	(41.3–42.1)	23.7	(23.4–24.0)	40.5	(40.1–40.9)
Others	34.6	(34.3–34.9)	20.8	(20.5–21.0)	32.4	(32.1–32.7)
*p*-value	< 0.001		< 0.001		< 0.001	
**Source of drinking water**						
Protected	30.4	(29.9–30.8)	21.1	(20.7–21.5)	29.2	(28.7–29.6)
Unprotected	39.3	(39.0–39.6)	22.0	(21.7–22.2)	37.2	(36.9–37.5)
*p*-value	< 0.001		< 0.001		< 0.001	
**Disposal of child stool**						
Safe	29.6	(29.2–30.0)	19.3	(19.0–19.6)	27.7	(27.3–28.1)
Unsafe	41.0	(40.7–41.4)	23.1	(22.8–23.4)	39.3	(38.9–39.6)
*p*-value	< 0.001		< 0.001		< 0.001	
**Place of residence**						
Urban	30.0	(29.6–30.5)	20.2	(19.7–20.6)	28.6	(28.1–29.0)
Rural	39.8	(39.5–40.1)	22.4	(22.2–22.6)	37.8	(37.5–38.1)
*p*-value	< 0.001		< 0.001		< 0.001	
**Region**						
North	34.0	(33.4–34.6)	20.3	(19.8–20.8)	30.2	(29.7–30.8)
Central	42.4	(42.0–42.9)	21.2	(20.8–21.6)	39.1	(38.7–39.6)
East	40.3	(39.8–40.9)	22.5	(22.0–23.0)	39.3	(38.7–39.8)
North east	32.4	(31.8–33.1)	15.1	(14.6–15.5)	25.0	(24.4–25.6)
West	34.4	(33.5–35.3)	26.6	(25.8–27.4)	36.7	(35.8–37.7)
South	28.6	(27.9–29.3)	20.2	(19.6–20.9)	27.3	(26.5–28.0)
*p*-value	< 0.001		< 0.001		< 0.001	
Total	36.9		21.7		36.4	

*p* values present the level of significance of Pearson’s chi-square statistics.

### Summary measures of different high-risk categories

Table 3 shows the prevalence of no risk, any single risk, and multiple high-risk fertility behaviour by background characteristics. Almost, 79% of children in the age bracket 0–11 months were born without high-risk fertility behaviour. However, any single high-risk fertility behaviour was present at birth in 27% of children between the ages of 48–59 months. A single high-risk category was experienced by almost 40% of the children of mothers aged 35–49, while multiple high-risk categories were experienced by 33% of the children. When a woman has no education, the prevalence of any single risk is 33%, and the prevalence of multiple risks is 14%. In the Muslim religion, 9% of infants were born with multiple risk groups, and 28% of children with any single risk. In the central region, 8% of children born to mothers with multiple high-risk fertility behaviours and 26% of children born to mothers in any single high-risk category. However, in the east region, 25% of children were born to mothers who fall into any single high-risk category, and 6% are born to mothers who fall into multiple high-risk categories.

**Table 3:** Prevalence of different high-risk fertility behaviour by sociodemographic characteristics among children aged 0–59 months, India NFHS-4,

Variables	No risk		Any single risk		Multiple risks	
	Prevalence	95% CI	Prevalence	95% CI	Prevalence	95% CI
**Current age of the child (months)**						
0–11	78.5	(78.1–79.0)	18.0	(17.6–18.5)	3.5	(3.2–3.7)
12–23	75.7	(75.2–76.2)	20.0	(19.6–20.5)	4.3	(4.0–4.5)
24–35	73.9	(73.4–74.4)	21.3	(20.9–21.8)	4.8	(4.6–5.0)
36–47	69.4	(68.9–69.9)	24.3	(23.9–24.8)	6.3	(6.0–6.6)
48–59	65.4	(64.9–65.9)	27.2	(26.7–27.7)	7.4	(7.1–7.7)
*p*-value	< 0.001		< 0.001		< 0.001	
**Gender of the child**						
Male	72.2	(71.8–72.5)	22.6	(22.3–22.9)	5.3	(5.1–5.4)
Female	72.8	(72.5–73.2)	21.9	(21.6–22.2)	5.3	(5.1–5.5)
*p*-value	< 0.01		< 0.001		0.483	
**Maternal age**						
15–24	80.7	(80.3–81.0)	18.6	(18.2–18.9)	0.8	(0.7–0.9)
25–34	75.4	(75.1–75.7)	21.4	(21.1–21.6)	3.2	(3.1–3.4)
35–49	27.2	(26.5–27.9)	40.2	(39.4–40.9)	32.7	(31.9–33.4)
*p*-value	< 0.001		< 0.001		< 0.001	
**Maternal education**						
No education	53.7	(53.2–54.1)	32.9	(32.4–33.3)	13.5	(13.1–13.8)
Primary	67.8	(67.2–68.5)	26.8	(26.2–27.4)	5.3	(5.0–5.6)
Secondary	80.0	(79.7–80.3)	18.2	(17.9–18.5)	1.8	(1.7–1.9)
Higher	89.7	(89.3–90.2)	9.6	(96.1–96.7)	0.6	(0.7–0.5)
*p*-value	< 0.001		< 0.001		< 0.001	
**Maternal body mass index**						
Under weight	69.5	(69.1–70.0)	24.6	(24.2–25.1)	5.8	(5.6–6.1)
Normal	72.6	(72.3–72.9)	22	(21.7–22.3)	5.4	(5.3–5.6)
Overweight	76.7	(76.1–77.3)	19.4	(18.9–19.9)	3.9	(3.6–4.2)
*p*-value	< 0.001		< 0.001		< 0.001	
**Contraceptive use**						
No	74	(73.7–74.3)	20.6	(20.3–20.9)	5.4	(5.3–5.6)
Yes	70.8	(70.5–71.2)	24.1	(23.7–24.4)	5.1	(4.9–5.3)
*p*-value	< 0.001		< 0.001		< 0.001	
**Household members**						
1–4	84.2	(83.8–84.6)	15.0	(14.6–15.3)	0.8	(0.7–0.9)
5–6	71.2	(70.8–71.6)	23.7	(23.4–24.1)	5.1	(4.9–5.3)
7 +	65.7	(65.3–66.1)	25.8	(25.4–26.2)	8.5	(8.2–8.7)
*p*-value	< 0.001		< 0.001		< 0.001	
**Wealth**						
Poor	66.2	(65.9–66.5)	26.4	(26.2–26.7)	7.4	(7.2–7.5)
Non poor	83.5	(83.1–83.8)	14.9	(14.6–15.3)	1.6	(1.5–1.7)
*p*-value	< 0.001		< 0.001		< 0.001	
**Religion**						
Hindu	73.8	(73.5–74.1)	22.0	(21.3–21.8)	4.7	(4.6–4.8)
Muslim	62.8	(62.1–63.5)	28.0	(27.1–28.4)	9.4	(9.0–9.8)
Others	77.2	(76.6–77.9)	19.0	(18.4–19.6)	3.7	(3.5–4.0)
*p*-value	< 0.001		< 0.001		< 0.001	
**Caste**						
Scheduled Caste/Tribes	69.7	(69.3–70.1)	24.3	(23.9–24.6)	6.0	(5.8–6.2)
Others	73.8	(73.5–74.1)	21.3	(21.0–21.5)	4.9	(4.8–5.0)
*p*-value	< 0.001		< 0.001		< 0.001	
**Source of drinking water**						
Protected	79.6	(79.2–80.0)	17.7	(17.3–18.0)	2.7	(2.6–2.9)
Unprotected	69.9	(69.6–70.1)	23.9	(23.7–24.2)	6.2	(6.1–6.3)
*p*-value	< 0.001		< 0.001		< 0.001	
**Child stool disposal**						
Safe	78.3	(77.9–78.6)	18.4	(18.1–18.8)	3.3	(3.1–3.4)
Unsafe	69.2	(68.9–69.5)	24.4	(24.2–24.7)	6.4	(6.2–6.6)
p-value	< 0.001		< 0.001		< 0.001	
**Place of residence**						
Urban	79.6	(79.2–80.0)	17.4	(17.0–17.8)	3.0	(2.9–3.2)
Rural	69.5	(69.2–69.8)	24.3	(24.1–24.6)	6.2	(6.1–6.4)
*p*-value	< 0.001		< 0.001		< 0.001	
**Region**						
North	73.9	(73.3–74.5)	21.5	(20.9–22.0)	4.7	(4.4–4.9)
Central	65.8	(65.4–66.2)	25.9	(25.5–26.3)	8.3	(8.1–8.6)
East	69.0	(68.4–69.5)	24.7	(24.2–25.2)	6.4	(6.1–6.7)
North east	73.7	(73.1–74.3)	20.5	(19.9–21.0)	5.8	(5.5–6.1)
West	78.8	(78.0–79.6)	18.4	(17.7–19.1)	2.8	(2.5–3.1)
South	81.5	(80.9–82.1)	17.2	(16.6–17.8)	1.3	(1.1–1.5)
*p*-value	< 0.001		< 0.001		< 0.001	
Total	72.47 (102,887)		22.25 (33,410)		5.27 (9,023)	

*p* values present the level of significance of Pearson’s chi-square statistics.

### Prevalence of stunting, wasting, and underweight by child age groups

**Figure 1:**
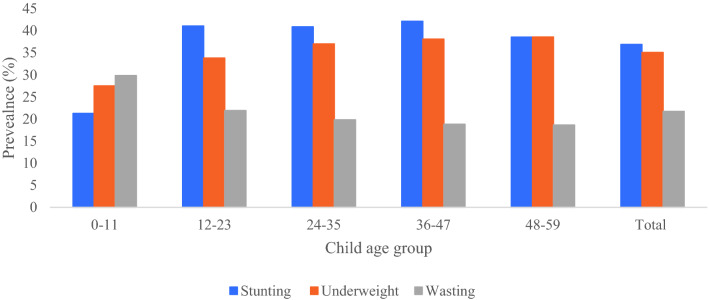
Prevalence of the stunting, wasting, and underweight among child age group 0–59 months, India, NFHS-2015–2016.

The prevalence of stunting, wasting, and underweight in children by age group is indicated in Fig. 1. The total prevalence of stunting was 37%, wasting 22%, and being underweight 35%. Stunting was found in 42% and 39% of underweight children aged 36–47 months, respectively. However, the age brackets 0–11 months had the highest rate of wasting.

### The relationship between high-risk fertility behaviour and stunting, wasting, and underweight

Table 4 shows the findings of the logistic regression of the stunting, wasting, and underweight with the sociodemographic variables. In the unadjusted model, the likelihood of stunting was 1.5 times (UOR:1.50, 95% CI 1.47–1.54) and the likelihood of multiple high-risk categories was 2 times (UOR:2.04, 95% CI 1.96–2.13) higher than the reference category no high risk births. The study adjusted socioeconomic, demographic, and household characteristics to assess the impact of high-risk fertility behaviour on the status of child malnutrition. The adjusted odds ratio shows that, compared to the no-risk category, the likelihood of stunting was about 1.3 times higher (OR:1.28, 95% CI 1.24–1.32), and the likelihood of multiple risks was approximately 1.6 times higher (OR:1.55, 95% CI 1.46–1.64). If we examine the likelihood of being underweight, it was roughly 1.2 times (OR:1.21, 95% CI 1.17–1.25) greater for any single risk and 1.4 times (OR:1.36, 95% CI 1.28–1.44) for multiple risks than reference category no risk. The nutritional status of male children was also more likely to be better than that of female children. The nutrition of children improved as maternal age, education, body mass index, and household wealth status significantly increased. According to the study, children exposed to unsafe soiled disposal were more likely to be stunted (OR:1.20; 95% CI 1.17–1.23), wasted (OR:1.08; 95% CI 1.05–1.12), and be underweight (OR:1.22, 95% CI 1.18–1.25). Surprisingly, children in rural areas were less likely than those living in urban areas to be stunted (OR:0.96; 95% CI 0.93–0.99), wasted (OR:0.95; 95% CI 0.92–0.99), and underweight (OR:0.0.91; 95% CI 0.88–0.94). In the current study, the likelihood of stunting was greater in the central region (OR:1.21; 95% CI 1.16–1.25), the west region (OR:1.15; 95% CI: 1.09–1.21), and the south region have (OR:0.95; 95% CI 0.90–0.99) than the reference category northern region of India.

**Table 4:** Unadjusted and adjusted odds ratios using logistic regression models investigating the relationship between mothers’ high-risk births behaviour and children's nutrition outcomes, India, (NFHS-4).

Variables	Stunting		Wasting		Underweight	
	Unadjusted OR(95% CI)	Adjusted OR (95% CI)	Unadjusted OR (95% CI)	Adjusted OR (95% CI)	Unadjusted OR (95% CI)	Adjusted OR (95% CI)
**Any high-risk category**						
No	1.05*** (1.03–1.08)	0.92*** (0.90–0.95)	1.0 2 (0.99–1.04)	0.99 (0.96–1.03)	1.03 (1.01–1.06)**	0.94*** (0.91–0.97)
Yes	Ref.	Ref.	Ref.	Ref.	Ref.	Ref.
**High-risk category**						
No risk	Ref.	Ref.	Ref.	Ref.	Ref.	Ref.
Single risk	1.50*** (1.47–1.54)	1.28*** (1.24–1.32)	0.99 (0.96–1.02)	0.99 (0.95–1.02)	1.41*** (1.37–1.44)	1.21*** (1.17–1.25)
Multiple risks	2.04*** (1.96–2.13)	1.55*** (1.46–1.64)	0.99 (0.95–1.05)	1.02 (0.95–1.09)	1.68*** (1.61–1.76)	1.36*** (1.28–1.44)
**Current age of the child (months)**						
0–11		Ref.		Ref.		Ref.
12–23		2.64*** (2.54–2.74)		0.69*** (0.66–0.71)		1.36*** (1.31–1.41)
24–35		2.65*** (2.55–2.76)		0.66*** (0.63–0.68)		1.64*** (1.58–1.70)
36–47		2.86*** (2.75–2.97)		0.58*** (0.56–0.61)		1.69*** (1.63–1.76)
48–59		2.40*** (2.31–2.50)		0.57*** (0.54–0.59)		1.70*** (1.63–1.76)
**Gender of the child**						
Male		Ref.		Ref.		Ref.
Female		0.90*** (0.88–0.92)		0.90*** (0.87–0.92)		0.93*** (0.91–0.95)
**Maternal age**						
15–24		Ref.		Ref.		Ref.
25–34		0.92*** (0.90–0.95)		1.03 (0.99–1.06)		0.96* (0.94–0.99)
35–49		0.86*** (0.82–0.90)		1.04 (0.99–1.10)		0.90*** (0.86–0.95)
**Maternal education**						
No education		Ref.		Ref.		Ref.
Primary		0.87*** (0.84–0.90)		0.93*** (0.89–0.97)		0.89*** (0.86–0.92)
Secondary		0.69*** (.67–0.71)		0.88*** (0.85–0.91)		0.72*** (0.70–0.74)
Higher		0.51*** (0.49–0.54)		0.84*** (0.79–0.89)		0.53*** (0.51–0.56)
**Maternal body mass index**						
Underweight		Ref.		Ref.		Ref.
Normal		0.79*** (0.77–0.81)		0.75*** (0.73–0.77)		0.62*** (0.60–0.63)
Overweight		0.61*** (0.58–0.63)		0.50*** (0.47–0.52)		0.40*** (0.38–0.42)
**Contraceptive use**						
No		Ref.		Ref.		Ref.
Yes		0.91*** (0.89–0.93)		0.98 (0.96–1.01)		0.95*** (0.93–0.98)
**Household members**						
1–4		Ref.		Ref.		Ref.
5–6		1.02 (0.99–1.05)		0.98 (0.94–1.01)		1.01 (0.98–1.04)
**7 + **		1.06*** (1.03–1.09)		0.90*** (0.87–0.93)		0.99 (0.97–1.03)
**Wealth**						
Poor		Ref.		Ref.		Ref.
Rich		0.70*** (0.68–0.72)		0.89*** (0.86–0.92)		0.72*** (0.69–0.74)
**Religion**						
Hindu		Ref.		Ref.		Ref.
Muslim		1.11*** (1.07–1.15)		0.93*** (0.89–0.97)		1.01 (0.98–1.05)
Others		0.95* (0.91–0.99)		0.92*** (0.87–0.96)		0.84*** (0.80–0.88)
**Caste**						
Scheduled Caste/Tribes		Ref.		Ref.		Ref.
Others		0.88*** (0.86–0.90)		0.89*** (0.86–0.91)		0.85*** (0.83–0.87)
**Source of drinking water**						
Protected		Ref.		Ref.		Ref.
Unprotected		0.99 (0.96–1.02)		0.98 (0.94–1.01)		1.01 (0.98–1.04)
**Disposal of child stool**						
Safe		Ref.		Ref.		Ref.
Unsafe		1.20*** (1.17–1.23)		1.08*** (1.05–1.12)		1.22*** (1.18–1.25)
**Place of residence**						
Urban		Ref.		Ref.		Ref.
Rural		0.96** (0.93–0.99)		0.95** (0.92–0.99)		0.91*** (0.88–0.94)
**Region**						
North		Ref.		Ref.		Ref.
Central		1.21*** (1.16–1.25)		1.02 (0.98–1.07)		1.25*** (1.21–1.30)
East		1.06* (1.02–1.10)		1.09*** (1.04–1.14)		1.24*** (1.19–1.29)
Northeast		0.82*** (0.78–0.86)		0.58*** (0.55–0.61)		0.57*** (0.54–0.60)
West		1.15*** (1.09–1.21)		1.44*** (1.36–1.52)		1.46*** (1.39–1.53)
South		0.95* (0.90–0.99)		1.09*** (1.03–1.15)		1.03 (0.98–1.08)

*OR:* Odds Ratio, *CI:* Confidence Interval.

Significant level: ***p < 0.001, **p < 0.01, *p < 0.05

### High-risk birth categories and prevalence of stunting, wasting, and underweight

The prevalence of stunting, wasting, and underweight is shown in Table 5 by the mother’s high-risk reproductive behaviour. Approximately, 39% of children born to women under the age of 18 were underweight, and around 41% were stunted. On the other hand, 41% of infants born during a birth interval of fewer than 24 months were underweight, and 43% of them were stunted. Around 41% of children were underweight and 48% were stunted when the birth order was more than four. Further, 52% of children were stunted and 47% were underweighted when they were born to mothers who were over 34 age and had more than four children. 53% of stunted and 48% of underweight children had those mothers age at birth > 34 years, a birth gap > 24 months, and a birth order > 4. When birth interval was more than 24 months and the birth order was larger than four, 56% of the kids were stunted and 50% were underweight.

**Table 5:** Prevalence of children nutrition outcomes by maternal high risk births conditions among currently married women children aged 0–59 months, India NFHS-4.

Variables	Stunting (%)		Wasting (%)		Underweight (%)	
	Prevalence	95% CI	Prevalence	95% CI	Prevalence	95% CI
**Births to mothers < 18 years**						
No	36.8	(36.5–37.0)	21.8	(21.5–22.0)	35	(34.7–35.2)
Yes	40.6	(38.9–42.3)	20.9	(19.5–22.3)	39	(37.3–40.6)
*p*-value	< 0.001		0.15		< 0.001	
**Births to mothers > 34 years**						
No	36	(35.8–36.2)	21.7	(21.5–21.9)	35.1	(34.9–35.4)
Yes	32.1	(30.4–33.8)	21.8	(20.2–23.3)	31.2	(29.5–32.9)
*p*-value	< 0.001		< 0.01		< 0.001	
**Births born < 24 months**						
No	36.2	(36.0–36.5)	21.7	(21.5–22.0)	34.5	(34.2–34.7)
Yes	43.3	(42.5–44.1)	21.7	(21.0–22.4)	40.9	(40.1–41.7)
*p*-value	< 0.001		0.46		< 0.001	
**Births with a birth order > 4**						
No	35.8	(35.5–36.1)	21.7	(21.5–21.9)	34.5	(34.2–34.7)
Yes	48.4	(47.6–49.2)	22	(21.3–22.7)	40.9	(40.1–41.7)
*p*-value	< 0.001		0.56		< 0.001	
**Age at birth < 18 years and birth interval < 24 months**						
No	36.9	(36.6–37.1)	21.7	(21.5–21.9)	35.1	(34.8–35.3)
Yes	51.2	(44.2–58.2)	23	(17.1–28.9)	41.9	(35.0–48.9)
*p*-value	0.368		0.71		0.24	
**Age at birth > 34 years and birth interval < 24 months**						
No	36.9	(36.7–37.1)	21.7	(21.5–21.9)	35.1	(34.8–35.3)
Yes	33.3	(25.3–41.3)	18.8	(12.2–25.4)	35.2	(27.2–43.3)
*p*-value	< 0.001		0.173		0.28	
**Age at birth > 34 years and birth order > 4**						
No	36.5	(36.2–36.8)	21.7	(21.5–21.9)	34.8	(34.5–35.0)
Yes	52.4	(50.9–53.9)	22.9	(21.7–24.2)	46.7	(45.2–48.1)
*p*-value	< 0.001		0.686		< 0.001	
**Age at birth > 34 years, birth internal < 24 months, and birth order > 4**						
No	36.9	(36.7–37.1)	21.7	(21.5–21.9)	35	(34.8–35.3)
Yes	53.1	(48.9–57.3)	21.1	(17.6–24.6)	48.2	(43.9–52.4)
*p*-value	< 0.001		0.753		< 0.001	
**Birth interval < 24 months and birth order > 4**						
No	36.4	(36.1–36.7)	21.7	(21.5–22.0)	34.7	(34.5–35.0)
Yes	55.9	(54.3–57.5)	21.2	(19.8–22.5)	49.5	(47.8–51.1)
*p*-value	< 0.001		0.346		< 0.001	

*p* values present the level of significance of Pearson’s chi-square statistics.

### The association between high-risk births and stunting, wasting, and underweight

The odds of different high-risk birth conditions and stunting, wasting, and underweight in children are shown in Table 6. The likelihood of stunting, wasting, and underweight among children born to mothers under the age of 18 years were 1.17 times (AOR:1.17; 95% CI 1.09–1.26), 0.86 times (AOR:0.86, 95% CI 0.79–0.94), and 1.11 times, respectively (AOR:1.11; 95% CI 1.03–1.20). Furthermore, likelihood of stunting risks were 1.28 times (AOR:1.28; 95% CI 1.23–1.33) and likelihood of underweight was 1.26 times (AOR:1.26; 95% CI 1.21–1.31) higher for births interval by fewer than 24 months compared to their respective counterparts. Among mothers with more than four birth orders, the likelihood of stunting were 1.19 times higher (AOR:1.19; 95% CI 1.15–1.24) and the likelihood of being underweight were 1.11 times higher (AOR:1.11; 95% CI 1.07–1.15). The likelihood of stunting was 1.26 times higher (AOR:1.26; 95% CI 1.18–1.34) and underweight was 1.09 times (AOR:1.09; 95% CI 1.02–1.17) higher if the mothers birth order was greater than four and the age at birth was greater than 34 years. The likelihood of stunting were 1.57 times (AOR:1.57; 95% CI 1.46–1.1.68) and underweight were 1.40 times (AOR:1.40; 95% CI 1.30–1.49) higher when the birth interval was 24 months and the birth order was greater than four.

**Table 6:** Unadjusted and adjusted odds ratios using binary logistic regression models investigating of the relationship between mother high-risk births behaviour and children's nutrition outcomes, India, (NFHS-4).

Variables	Stunting		Wasting		Underweight	
	Unadjusted OR (95% CI)	Adjusted OR (95% CI)	Unadjusted OR (95% CI)	Adjusted OR (95% CI)	Unadjusted OR (95% CI)	Adjusted OR (95% CI)
**Births to mothers < 18 years**						
No	Ref.	Ref.	Ref.	Ref.	Ref.	Ref.
Yes	1.23*** (1.14–1.32)	1.17*** (1.09–1.26)	0.94 (0.86–1.02)	0.86*** (0.79–0.94)	1.22*** (1.14–1.31)	1.11* (1.03–1.20)
**Births to mothers > 34 years**						
No	Ref.	Ref.	Ref.	Ref.	Ref.	Ref.
Yes	0.74*** (0.68–0.81)	0.95 (0.88–1.04)	0.87** (0.79–0.96)	0.99 (0.89–1.10)	0.72*** (0.66–0.78)	0.97 (0.89–1.06)
**Births born < 24 months**						
No	Ref.	Ref.	Ref.	Ref.	Ref.	Ref.
Yes	1.32*** (1.27–1.37)	1.28*** (1.23–1.33)	1.02 (0.97–1.06)	1.01 (0.97–1.06)	1.32*** (1.27–1.37)	1.26*** (1.21–1.31)
**Births with a birth order > 4**						
No	Ref.	Ref.	Ref.	Ref.	Ref.	Ref.
Yes	1.55*** (1.49–1.61)	1.19*** (1.15–1.24)	1.01 (0.97–1.06)	0.98 (0.93–1.03)	1.41*** (1.36–1.46)	1.11*** (1.07–1.15)
**Age at birth < 18 years and birth interval < 24 months**						
No	Ref.	Ref.	Ref.	Ref.	Ref.	Ref.
Yes	1.61*** (1.22–2.14)	1.31 (0.98–1.74)	0.94 (0.66–1.33)	0.96 (0.68–1.37)	1.19 (0.88–1.58)	0.95 (0.71–1.28)
**Age at birth > 34 years and birth interval < 24 months**						
No	Ref.	Ref.	Ref.	Ref.	Ref.	Ref.
Yes	0.85 (0.59–1.21)	0.99 (0.68–1.43)	0.73 (0.46–1.15)	0.89 (0.56–1.42)	0.82 (0.56–1.18)	1.06 (0.72–1.55)
**Age at birth > 34 years and birth order > 4**						
No	Ref.	Ref.	Ref.	Ref.	Ref.	Ref.
Yes	1.63*** (1.53–1.73)	1.26*** (1.18–1.34)	0.98 (0.91–1.06)	0.98 (0.90–1.07)	1.34*** (1.26–1.42)	1.09*** (1.02–1.17)
**Age at birth > 34 years, birth internal < 24 months, and birth order > 4**						
No	Ref.	Ref.	Ref.	Ref.	Ref.	Ref.
Yes	1.86*** (1.56–2.20)	1.31** (1.10–1.57)	0.97 (0.78–1.19)	1.02 (0.82–1.27)	1.57*** (1.32–1.86)	1.20** (1.00–1.44)
**Birth interval < 24 months and birth order > 4**						
No	Ref.	Ref.	Ref.	Ref.	Ref.	Ref.
Yes	2.09*** (1.96–2.23)	1.57*** (1.46–1.68)	1.04 (0.96–1.12)	1.04 (0.95–1.13)	1.81*** (1.69–1.93)	1.40*** (1.30–1.49)

Adjusted models were controlled for children age, child sex, mother education, mother body mass index, contraceptive use, household’s members, wealth, religion, caste, source of drinking water, place of residence, region.

*OR:* Odds Ratio, *CI:* Confidence Interval.

Significant level: ***p < 0.001, **p < 0.01, *p < 0.05

## Discussion

The current analysis using the nationally-representative data of Indian women found that high-risk fertility behaviors are highly frequent in India. A proportion of 35% of married women had at least one of the high-risk fertility behaviors confirming that it is alarmingly common in this South Asian country. This included 9.4% of women having a birth interval of less than 24 months and 8.7% of women having a birth order of more than four. The findings are in line with studies that documented that around 46% of women in South Asia were married before the age of 18 years^41^. Similarly, a higher rate of teenage pregnancy (35%) was reported in Bangladesh^42^. Low birth interval (less than 2 years) was found to be highly prevalent in India and Nepal^43^. The same study also reported the prevalence of birth order of more than three children as high as 12% in Nepal and Bangladesh.

Furthermore, despite major improvements in indicators of children’s health in the country over the last decade, current findings reveal that stunting (36.9%), wasting (21.7%), and underweight (36.4%) remain major concerns among Indian children. Narayan et al.^43^ suggest that in India, with nearly half of its child population being malnourished, there remains an urgent need for effective interventions by addressing the issues and challenges of current policies and programs on reducing child malnutrition^44^. In this context, maternal characteristics and reproductive behaviours have been found to significantly influence child health^45,46^. For example, women have unwanted pregnancies at later ages, and the behaviours associated with them represent the risk factors for premature birth, low birth weight, and child malnutrition^47^. Previous research has further shown that maternal age of less than 18 years and short intervals are also associated with prematurity and low birth weight, which results in child stunting^48–50^, as well as under-five mortality^51^. Furthermore, evidence from low and middle-income countries suggests that women who become pregnant soon after marriage at their younger ages are prone to have under-nourished or malnourished children^52–54^.

Consistent with these findings^24,43,51^, after adjusting for relevant covariates, our analyses found significant positive associations between single as well as multiple high-risk fertility behaviours in mothers with childhood stunting and underweight. These results provide a critical context for the prior studies in India and other developing countries reporting the increased risk of infant and under-five mortality^55–59^. As evident from past research, the social and health-related vulnerabilities among mothers with high-risk fertility behaviour such as early and late pregnancy, low birth interval, and high birth order that result in delivery of unhealthy children include increased rates of poverty and patriarchal gender norms in the community, which lead to maternal depression and malnutrition^54,56,60,61^. On the other hand, the biological factors that have a great influence on the observed associations include pregnancy-induced hypertension, iron-deficiency anaemia, prematurity, intrauterine growth retardation, mother-fetus competition for scarce nutrients, and congenital abnormalities^57,62–64^.

Furthermore, the current findings on multiple high-risk fertility behaviours and their association with adverse child nutritional outcomes of stunting and underweight could also be explained by lack of or limited access to health care leading to lower use of antenatal care, incomplete vaccination for infants, unskilled or semi-skilled delivery care for the child including the higher exposure of children to infectious pathogens, insufficient nutrient intake of mothers and inadequate feeding practices^65–67^. In this regard, high-risk fertility is also related to poor mental and physical health of mothers, pregnancy complications, and, in some cases, maternal mortality, all of which increase the chances of negative infant and childhood health conditions^68–70^. A recent study in Sri Lanka also linked the low socioeconomic status to a double burden of maternal and child malnutrition^71^.

Another finding of the present study is that the association of category of any or multiple high-risk fertility appeared to be significant with stunting and underweight but not with wasting. This suggests the need for further investigation. Considering the findings of the current analyses, which are in line with previous observations, health interventions based on specific high-risk fertility behaviour would help ensure the maternal and child health of those who are at higher levels of socioeconomic and biological vulnerabilities.

There are several limitations of the study to be noted. The exposure variables of high-risk fertility behaviour are based on self-report, resulting in recall bias. Also, importantly the design of the current analysis is cross-sectional, which does not allow inferring causality in the observed associations, suggesting the need for prospective investigation to evaluate the effects of high-risk fertility on children's health. Nevertheless, since the birth of the child and birth interval occurred before the collection of data assessing the child's nutritional status, ordering of the risk exposure to the child's health outcome can be assumed. In spite of these limitations, there are several strengths of the study. The study used data from a nationally representative sample of married women aged 15–49 years old, covering rural and urban areas with many subjects. Also, our study brings to important light information that could serve as a basis to reduce the risk of chronic child under-nutrition in India. Our results may also be relevant in other poor-resource settings where child malnutrition is common. They also may be of interest to clinicians assessing the nutritional problems of children relating it to the maternal fertility behaviour.

## Conclusion

A mother's high-risk fertility behaviour is an important risk factor for higher risk of stunting and being underweight among children under 5 years. Our findings underscore the calls for avoiding high-risk fertility largely in the form of too early or too late childbearing patterns, the higher number of total live births, and short birth spacing in order to reduce the risk of chronic under-nutrition among children under 5 years of age. The study also suggests that mothers’ receipt of appropriate health services and adequate feeding practices for children should be ensured, which may, in turn, facilitate improved maternal and child health. Further investigation of the causal link between high-risk fertility and nutritional outcomes of children will be critical to developing interventions to improve the nutritional status of children, which is a public health priority.

## Data Availability

The datasets used in this study can be found in the Demographic Health Surveys (DHS) repository https://dhsprogram.com.

## References

[1] Bertrand NAS (2018). Inequalities in infant malnutrition between rural and urban areas in Cameroon: a Blinder–Oaxaca decomposition. Afr. J. Econ. Rev..

[2] Sunil TS, Sagna M (2015). Decomposition of childhood malnutrition in C ambodia. Matern. Child Nutr..

[3] UNICEF, WHO W. *Levels and Trends in Child Malnutrition: Key Findings of the 2019 Edition of the Joint Child Malnutrition Estimates*. World Health Organization (2021).

[4] De P, Chattopadhyay N (2019). Effects of malnutrition on child development: Evidence from a backward district of India. Clin Epidemiol Glob Health.

[5] Pelletier DL, Frongillo EA, Schroeder DG, Habicht JP (1995). The effects of malnutrition on child mortality in developing countries. Bull World Health Organ.

[6] Kandala N-B, Madungu TP, Emina JB, Nzita KP, Cappuccio FP (2011). Malnutrition among children under the age of five in the democratic republic of Congo (DRC): Does geographic location matter?. BMC Public Health.

[7] Malnutrition in Children. UNICEF DATA (2021).

[8] World Health Organization. Maternal mortality in 2005: Estimates developed by WHO, UNICEF, UNFPA, and the World Bank (2020).

[9] Gragnolati M, Bredenkamp C, Shekar M, Das Gupta M, Lee Y-K (2006). India’s Undernourished Children: A Call for Reform and Action Health, Nutrition, and Population.

[10] IIPS (2017). National Family Health Survey (NFHS-4), 2015–2016.

[11] UNDP. *The next frontier Human development and the Anthropocene*. (2020).

[12] GH, I. Global Hunger Index‐Peer‐Reviewed Annual Publication Designed to Comprehensively Measure and Track Hunger at the Global, Regional, and Country Levels.0.

[13] Saha S, Singh R (2021). Child Malnutrition in India: A Systemic Failure.

[14] Singh S, Srivastava S, Upadhyay AK (2019). Socio-economic inequality in malnutrition among children in India: An analysis of 640 districts from National Family Health Survey (2015–2016). Int. J. Equity Health.

[15] Sahu SK (2015). Malnutrition among under-five children in India and strategies for control. J. Nat. Sci. Biol. Med..

[16] Chambers, R. & Von Medeazza, G. Sanitation and stunting in India: Undernutrition’s blind spot. *Econ. Polit. Week*. 15–18 (2013).

[17] Murarkar S (2020). Prevalence and determinants of undernutrition among under-five children residing in urban slums and rural area, Maharashtra, India: A community-based cross-sectional study. BMC Public Health.

[18] Sinha RK, Dua R, Bijalwan V, Rohatgi S, Kumar P (2018). Determinants of stunting, wasting, and underweight in five high-burden pockets of four Indian States. Indian J. Commun. Med..

[19] Ansuya (2018). Risk factors for malnutrition among preschool children in rural Karnataka: A case-control study. BMC Public Health.

[20] Dewey KG, Cohen RJ (2007). Does birth spacing affect maternal or child nutritional status? A systematic literature review. Matern. Child Nutr..

[21] Fenske N, Burns J, Hothorn T, Rehfuess EA (2013). Understanding child stunting in India: A comprehensive analysis of socio-economic, nutritional and environmental determinants using additive quantile regression. PLoS ONE.

[22] Suri, S. Anaemia and malnutrition: A vicious cycle leading to child growth failure. (2021).

[23] Barman P, Sahoo H (2021). Sex preference in India: Trends, patterns and determinants. Child Youth Serv. Rev..

[24] Rahman M (2016). Association between order of birth and chronic malnutrition of children: A study of nationally representative Bangladeshi sample. Cad. Saude Publica..

[25] Reichman NE, Corman H, Noonan K, Schwartz-Soicher O (2010). Effects of prenatal care on maternal postpartum behaviors. Rev. Econ. Househ..

[26] Kumar G (2019). Utilisation, equity and determinants of full antenatal care in India: Analysis from the National Family Health Survey 4. BMC Pregnancy Childbirth.

[27] Wemakor A, Garti H, Azongo T, Garti H, Atosona A (2018). Young maternal age is a risk factor for child undernutrition in Tamale metropolis. Ghana. BMC Res. Notes.

[28] Yu SH, Mason J, Crum J, Cappa C, Hotchkiss DR (2016). Differential effects of young maternal age on child growth. Global Health Action.

[29] Beri R, Upadhyaya AS, Kolås Å (2022). Food Governance in India: Rights Security and Challenges in the Global Sphere.

[30] Mtumwa AH, Paul E, Vuai SAH (2016). Determinants of undernutrition among women of reproductive age in Tanzania mainland. South Afr. J. Clin. Nutr.

[31] Tessema ZT, Tamirat KS (2020). Determinants of high-risk fertility behavior among reproductive-age women in Ethiopia using the recent Ethiopian demographic health survey: A multilevel analysis. Trop. Med. Health.

[32] Howlader MH (2022). Determinants associated with high-risk fertility behaviours among reproductive aged women in Bangladesh: A cross-sectional study. Reprod. Health.

[33] Tamirat KS, Tesema GA, Tessema ZT (2021). Determinants of maternal high-risk fertility behaviors and its correlation with child stunting and anemia in the East Africa region: A pooled analysis of nine East African countries. PLoS ONE.

[34] Das M, Verma M, Sahoo SS, Gupta M (2022). Regional water availability and WASH indicators as predictors of malnutrition in under-5 children: Analysis of the National Family Health Survey, India (2015–2016). J. Trop. Pediatr..

[35] World Health Organization (1995). Physical Status : The Use and Interpretation of Anthropometry.

[36] Mozumder AB, Barkat-E-Khuda, Kane TT, Levin A, Ahmed S (2000). The effect of birth interval on malnutrition in Bangladeshi infants and young children. J. Biosoc. Sci..

[37] Rahman M (2018). Maternal high-risk fertility behavior and association with chronic undernutrition among children under age 5 year in India, Bangladesh, and Nepal: Do poor children have a higher risk?. Nutrition.

[38] Fenske N, Burns J, Hothorn T, Rehfuess EA (2013). Understanding child stunting in India: A comprehensive analysis of socio-economic, nutritional and environmental determinants using additive quantile regression. PLoS ONE.

[39] WHO (2006). Core Questions on Drinking Water and Sanitation for Household Surveys.

[40] Loaiza E, Wong S (2012). Marrying too young. End child marriage.

[41] NIPORT. *Bangladesh Demograph and Health Survey*. Bangladesh Demographic and Health Survey 2017–2018 (2020).

[42] Rahman M (2018). Maternal high-risk fertility behavior and association with chronic undernutrition among children under age 5 year in India, Bangladesh, and Nepal: do poor children have a higher risk?. Nutrition.

[43] Narayan J, John D, Ramadas N (2019). Malnutrition in India: Status and government initiatives. J. Public Health Policy.

[44] Rana MJ, Cleland J, Sekher TV, Padmadas SS (2021). Disentangling the effects of reproductive behaviours and fertility preferences on child growth in India. Popul. Stud..

[45] Paul VK (2011). Reproductive health, and child health and nutrition in India: Meeting the challenge. The Lancet.

[46] Neal S, Channon AA, Chintsanya J (2018). The impact of young maternal age at birth on neonatal mortality: Evidence from 45 low and middle income countries. PLoS ONE.

[47] Conde-Agudelo A, Rosas-Bermudez A, Castaño F, Norton MH (2012). Effects of birth spacing on maternal, perinatal, infant, and child health: A systematic review of causal mechanisms. Stud. Fam. Plann..

[48] Conde-Agudelo A, Belizán JM, Norton MH, Rosas-Bermúdez A (2005). Effect of the interpregnancy interval on perinatal outcomes in Latin America. Obstet. Gynecol..

[49] Da Vanzo J, Hale L, Razzaque A, Rahman M (2008). The effects of pregnancy spacing on infant and child mortality in Matlab, Bangladesh: How they vary by the type of pregnancy outcome that began the interval. Popul. Stud..

[50] Amir-ud-Din, R., Naz, L., Rubi, A., Usman, M. & Ghimire, U. Impact of high-risk fertility behavior on under-five mortality in Asia and Africa: Evidence from demographic and health surveys. (2020) 10.21203/rs.3.rs-29205/v1. 10.1186/s12884-021-03780-y PMC8088561 33933011

[51] Delprato M, Akyeampong K (2017). The effect of early marriage timing on women’s and children’s health in Sub-Saharan Africa and Southwest Asia. Ann. Glob. Health.

[52] Wulandari UR, Budihastuti UR, Pamungkasari EP (2017). Analysis of Life-course factors influencing growth and development in children under 3 years old of early marriage women in Kediri. J. Matern. Child Health.

[53] Raj A (2010). The effect of maternal child marriage on morbidity and mortality of children under 5 in India: Cross sectional study of a nationally representative sample. BMJ (Online).

[54] Fotso JC, Cleland J, Mberu B, Mutua M, Elungata P (2013). Birth spacing and child mortality: An analysis of prospective data from the nairobi urban health and demographic surveillance system. J. Biosoc. Sci..

[55] Sonneveldt E, Decormier Plosky W, Stover J (2013). Linking high parity and maternal and child mortality: What is the impact of lower health services coverage among higher order births?. BMC Public Health.

[56] Conde-Agudelo A, Rosas-Bermúdez A, Kafury-Goeta AC (2007). Effects of birth spacing on maternal health: A systematic review. Am. J. Obstet. Gynecol..

[57] Singh R, Tripathi V (2013). Maternal factors contributing to under-five mortality at birth order 1–5 in India: A comprehensive multivariate study. Springerplus.

[58] Mayor S (2004). Pregnancy and childbirth are leading causes of death in teenage girls in developing countries. BMJ (Clin. Res. Ed.).

[59] Adhikari R (2010). Demographic, socio-economic, and cultural factors affecting fertility differentials in Nepal. BMC Pregnancy Childbirth.

[60] Das B, Tarai D (2011). Decision-making and fertility behaviour: A comparative analysis of scheduled caste and scheduled tribe women in Odisha. Soc. Change.

[61] Stewart CP (2007). Preterm delivery but not intrauterine growth retardation is associated with young maternal age among primiparae in rural Nepal. Matern. Child Nutr..

[62] Rahman LA, Hairi NN, Salleh N (2008). Association between pregnancy induced hypertension and low birth weight: A population based case-control study. Asia Pac. J. Public Health.

[63] Parveen N, Haider G, Shaikh IA, Ujjan ID (2009). Presentation of predisposing factors of pregnancy induced hypertension at Isra University Hospital, Hyderabad. J. Liaquat Univ. Med. Health Sci..

[64] Larrea C, Kawachi I (2005). Does economic inequality affect child malnutrition? The case of ecuador. Soc. Sci. Med..

[65] Brennan L, McDonald J, Shlomowitz R (2004). Infant feeding practices and chronic child malnutrition in the Indian states of Karnataka and Uttar Pradesh. Econ. Hum. Biol..

[66] Rahman MM (2015). Is Unwanted birth associated with child malnutrition in Bangladesh?. Int. Perspect. Sex. Reprod. Health.

[67] Santos DS, Santos DN, De Cássia Ribeiro Silva R, Hasselmann MH, Barreto ML (2011). Maternal common mental disorders and malnutrition in children: A case-control study. Soc. Psychiatr. Psychiatr. Epidemiol..

[68] Silveira KBR, Alves JFR, Ferreira HS, Sawaya AL, Florêncio TMMT (2010). Association between malnutrition in children living in favelas, maternal nutritional status, and environmental factors. J. Pediatr..

[69] Harpham T, Huttly S, De Silva MJ, Abramsky T (2005). Maternal mental health and child nutritional status in four developing countries. J. Epidemiol. Commun. Health.

[70] Shinsugi C (2019). Double burden of maternal and child malnutrition and socioeconomic status in urban Sri Lanka. PLoS ONE.

